# Prediction of Minimal Inhibitory Concentration of Meropenem Against *Klebsiella pneumoniae* Using Metagenomic Data

**DOI:** 10.3389/fmicb.2021.712886

**Published:** 2021-08-23

**Authors:** Rundong Tan, Anqi Yu, Ziming Liu, Ziqi Liu, Rongfeng Jiang, Xiaoli Wang, Jialin Liu, Junhui Gao, Xinjun Wang

**Affiliations:** ^1^Shanghai Biotecan Pharmaceuticals Co., Ltd., Shanghai, China; ^2^Shanghai Zhangjiang Institute of Medical Innovation, Shanghai, China; ^3^Medical Information Engineering, Department of Medical Information, Harbin Medical University, Harbin, China; ^4^Department of Biostatistics, School of Global Public Health, New York University, New York, NY, United States; ^5^Department of Critical Care Medicine, Ruijin Hospital, School of Medicine, Shanghai Jiao Tong University, Shanghai, China; ^6^Translational Medical Center for Stem Cell Therapy, Shanghai East Hospital, School of Medicine, Tongji University, Shanghai, China

**Keywords:** *Klebsiella pneumoniae*, minimum inhibitory concentration, meropenem, XGBoost, deep neural network

## Abstract

Minimal inhibitory concentration (MIC) is defined as the lowest concentration of an antimicrobial agent that can inhibit the visible growth of a particular microorganism after overnight incubation. Clinically, antibiotic doses for specific infections are determined according to the fraction of MIC. Therefore, credible assessment of MICs will provide a physician valuable information on the choice of therapeutic strategy. Early and precise usage of antibiotics is the key to an infection therapy. Compared with the traditional culture-based method, the approach of whole genome sequencing to identify MICs can shorten the experimental time, thereby improving clinical efficacy. *Klebsiella pneumoniae* is one of the most significant members of the genus *Klebsiella* in the Enterobacteriaceae family and also a common non-social pathogen. Meropenem is a broad-spectrum antibacterial agent of the carbapenem family, which can produce antibacterial effects of most Gram-positive and -negative bacteria. In this study, we used single-nucleotide polymorphism (SNP) information and nucleotide *k*-mers count based on metagenomic data to predict MICs of meropenem against *K. pneumoniae*. Then, features of 110 sequenced *K. pneumoniae* genome data were combined and modeled with XGBoost algorithm and deep neural network (DNN) algorithm to predict MICs. We first use the XGBoost classification model and the XGBoost regression model. After five runs, the average accuracy of the test set was calculated. The accuracy of using nucleotide *k*-mers to predict MICs of the XGBoost classification model and XGBoost regression model was 84.5 and 89.1%. The accuracy of SNP in predicting MIC was 80 and 81.8%, respectively. The results show that XGBoost regression is better than XGBoost classification in both nucleotide *k*-mers and SNPs to predict MICs. We further selected 40 nucleotide *k*-mers and 40 SNPs with the highest correlation with MIC values as features to retrain the XGBoost regression model and DNN regression model. After 100 and 1,000 runs, the results show that the accuracy of the two models was improved. The accuracy of the XGBoost regression model for *k*-mers, SNPs, and *k*-mers & SNPs was 91.1, 85.2, and 91.3%, respectively. The accuracy of the DNN regression model was 91.9, 87.1, and 91.8%, respectively. Through external verification, some of the selected features were found to be related to drug resistance.

## Introduction

*Klebsiella pneumoniae* is a member of thew enterobacter *Klebsiella*; it is a Gram-negative bacterium that causes one-third of all Gram-negative infections (Navon-Venezia et al., 2017). Over the past two decades, *K. pneumoniae* has undergone complex evolution, with the emergence of many high-risk, highly infectious sequence types, resulting in the sustained global spread of *K. pneumoniae* (Navon-Venezia et al., 2017). In addition to widespread transmission, the increase in drug resistance in *K. pneumoniae* is also an important issue. Many studies and reports indicate that antimicrobial resistance (AMR) strains of *K. pneumoniae* have increased at an alarming rate in recent years (Long et al., 2017; Navon-Venezia et al., 2017).

Carbapenem antibiotics play an important role in the treatment of severe infections of drug-resistant Enterobacteriaceae, and the increase of drug resistance of *K. pneumoniae* and the emergence and spread of drug-resistant strains pose a serious threat to public health (Spagnolo et al., 2014). In fact, carbapenem antibiotic resistance in *K. pneumoniae* has emerged many years ago and has spread widely around the world (Spagnolo et al., 2014). Recent studies have shown that the resistance rates of *K. pneumoniae* to aztreonam, ceftazidime, ciprofloxacin, cefotaxime, cefepime and imipenem are more than 50% (Effah et al., 2020). Meropenem has good *in vitro* anti-*K. pneumoniae* properties and is likely to have optimal bactericidal efficacy for the treatment of *K. pneumoniae* (Baldwin et al., 2008).

Meropenem belongs to the carbapenem class of antibiotics and is one of the widely used antibiotics for the treatment of *K. pneumoniae* infections, with broad-spectrum *in vitro* resistance to both Gram-positive and Gram-negative pathogens (Navon-Venezia et al., 2017). It readily penetrates the cell walls of most Gram-negative and -positive bacteria to reach its target penicillin-binding protein (PBPS) and exhibits stability to hydrolysis by most β-lactamases, including penicillinases and cephalosporinases produced by Gram-positive and Gram-negative bacteria (Navon-Venezia et al., 2017).

In addition to the selection of antimicrobial agents, the timing and dosage of effective antimicrobial agents are also very important. In general, treatment is most effective when effective antibiotics are administered early. In a study of patients with infectious shock, there was a strong relationship between time to effective antimicrobial drug onset and in-hospital mortality (corrected ratio 1.119 per hour delay) (Pesesky et al., 2016). Neither too high nor too low a dose of antibiotics is the optimal treatment regimen: too high may result in increased resistance to *K. pneumoniae*, and too low will not achieve the desired effect of treatment with antibiotics. The minimum inhibitory concentration (MIC) indicates the appropriate dosage of antibiotics. MIC is an important index to measure both the effectiveness of antimicrobial agents and bacterial resistance to drugs.

Treatment with the optimal dose of effective antibiotics as soon as possible after the infection is the key to curing *K. pneumoniae* infection. Therefore, the time required to determine the MIC is an important factor to determine whether antibiotics can be used in the early stage of infection. There are many traditional methods of MIC determination, such as spatial gas chromatography methods for antimicrobial screening, electronic testing methods, and traditional petri dish measurement methods. However, traditional methods often take 18 to 24 h or even more. In order to meet the demand for antibiotic therapy, we need to find newer, faster, and more accurate techniques for detecting the MIC of antibiotics.

In recent years, many researchers used machine learning methods to build models that can predict MIC value more quickly and accurately (Li et al., 2016, 2017; Eyre et al., 2017; Nguyen et al., 2018; Pataki et al., 2020). These papers presented the methods and models that were used to predict the MICs of *K. pneumoniae* (Nguyen et al., 2018), antibiotic moldus of *Neisseria gonorrhoeae* (Eyre et al., 2017), *Streptococcus pneumoniae* (Li et al., 2016), non-typhoid *Salmonella* (Nguyen et al., 2019), and *Escherichia coli* (Pataki et al., 2020).

A previous study has built XGBoost machine learning models to predict MICs for a comprehensive population-based collection of clinical isolates of *K. pneumoniae*, which was able to rapidly predict MICs for 20 antibiotics with an average accuracy of 92% (Nguyen et al., 2018). According to this, our study is dedicated to constructing models that can predict MICs for Meropenem treatment of *K. pneumoniae* more accurately and analyzing features that are highly correlated with MIC prediction and externally validating these features.

In this study, we first obtained single-nucleotide polymorphism (SNP) information and nucleotide *k*-mers (*k* = 6, 8, 10) counting information based on metagenomic data of *K. pneumoniae* sequence analysis and then trained the dataset with three machine learning and deep learning methods – XGBoost classification method, XGBoost regression method, and deep neural network (DNN) regression method – and finally compare the prediction results of the three methods and select the features that are highly related to MIC to construct a new prediction model to achieve higher prediction accuracy.

## Materials and Methods

### Data Collection

Two types of data were included in our study: *K. pneumoniae* metagenomic sequences, and the related MIC values of the antibiotic meropenem. The metagenomic data were pre-processed as tables of *k*-mers and SNPs for further model construction and prediction. Sequenced *K. pneumoniae* genome data used in this study can be downloaded *via* BioProject with access numbers PRJNA376414, PRJNA386693, and PRJNA396774. We collected data related to the antibiotic meropenem with complete sequence information and correct scaffold assembly, and finally, the 110 genome was involved in the study. The SRA access number for each genome is shown in the supplementary table.

HS11286^1^ was selected to be our reference genome for SNP calling. The table file with SRA ID and MIC values for meropenem was downloaded from the supplementary materials attached from Nguyen et al. (2018).

For sequence data, the fastq-dump tool SRA Toolkit was used (with -I –split-files parameters). SPAdes (Bankevich et al., 2012) was then used to (with −1, −2 and -o parameters) assemble the pair the end sequence for each sample. Finally, the assembled scaffold.fasta files were mapped to the reference genome to obtain *k*-mers and SNP information.

### Data Pre-processing

#### Nucleotide *k*-mers

In the study, 110 assembled genome scaffold files were processed to produce matrices of *k*-mers features. For each genome, we cut the scaffold sequences starting from the first nucleotide with 6-, 8-, and 10-nucleotide window lengths, respectively. For the following cuts, starting points of the windows move forward with one nucleotide each time until the sequence ends. Finally, a matrix with 110 rows and 559,494 columns of 6, 8, and 10 length nucleotide fragments were created for model training.

#### Calling SNPs

According to studies by Yang et al. (2018, 2019), SNPs resistant to *Mycobacterium tuberculosis* were used as features for prediction.

We extracted SNPs from the whole gene to find the resistant SNPs. For SNP calling, the raw 110 *K. pneumoniae* metagenomic samples were mapped to the HS11286 (“see text footnote 1”) reference genome with single end reads mode, and then reads of the 110 genome samples were mapped to the reference genome using samtoolsv1.9 (Bonfield et al., 2021) and resulting in 110.vcf files. Further filtering was conducted using bcftools v1.10 (Li, 2011) (with parameters %QUAL ≥ 50 & DP ≥ 20). Finally, a combined matrix of the combined SNPs with 110 rows and 164,138 columns was obtained. The columns of the matrix represent the concatenation of the SNP positions compared to the reference genome, where a sample with a mutation at that position was marked as 1 and those without mutations were marked as 0.

### EXtreme Gradient Boosting (XGBoost) Model Development

#### XGBoost

EXtreme Gradient Boosting (XGBoost) algorithm is an optimized distributed implementation of gradient boosted decision trees, designed for computational speed and higher performance. Since its initial release in 2014 (Chen and Guestrin, 2016), in the past few years, XGBoost has been applied to a number of biomedical problems.

As an implement machine learning algorithm under the gradient boosting framework, the starting point of XGBoost is decision trees. However, here, each tree is fitted to the residuals (prediction errors) of the previous tree in order to gradually minimize the deviations between the model and the observed target data. This is done by giving more weight to the poorly modeled cases. In contrast to the Random Forest model, the trees are thus not independent of each other. Besides the different random samples, this is additionally achieved by the fact that not all predictors are available for selection at each branching, but only a randomly chosen subset, and get exceptionally high performance for regression as well as classification tasks. Classification trees are used to identify the class/category within which the input variables would most likely fall, while regression algorithms are suitable for continuous variables, and the tree is used to predict the value.

XGBoost algorithm has gradient boosting at its core. However, unlike simple gradient boosting algorithms, the XGboost model takes a parallelization approach in the process of sequential addition of the weak learners, whereby proper utilization of the CPU core of the machine is utilized, leading to greater speed and performance (Santhanam, 2016). Moreover, it is a distributed and scalable computing method that is available for large datasets.

Moreover, one benefit of the gradient boosting model is that for different loss functions, new algorithms are not required to be derived; it is enough that a suitable loss function be chosen and then incorporated with the gradient boosting framework.

#### Model Training

We used XGBoost to train both classification and regression models, respectively; several predict models were built depending on data type.

For *k*-mers data, the occurrence times of each *k*-mer in each sample were counted, and we used all possible segments as features and mapped the number of *k*-mers to [0, 1] with Min–Max normalization. For SNPs data, features were characterized by binary number as zeros and ones of all mutation sites. The data were divided into training and test set as 8:2.

Our XGBoost models were set as tree-based structure (with ***booster* = *“gbtree”***), and GridsearchCV was applied for hyperparameter tuning. In order to prevent the XGBoost training process from generating too many trees, which causes the machine learning model to eventually overfit, we use fivefold cross-validation to select the most appropriate number of iterations; the value of booster_round is used as the num of XGBoost booster_round parameter, which is brought into the model training. Also, considering that our dataset is on the small side, using cross-validation also allows training with as much data as possible.

We first trained the XGboost multi-classification model, with the objective parameter ***Multi: Softmax***. Input samples are fed into the generated XGBoost tree, and the leaf to which the sample belongs is found in each tree; the belonging weight is then added to obtain the predictions. As it is a multiclass classification model, we set 17 categories as classification labels to train the model, with a minimum MIC value of 0 and a maximum MIC value of 16, equally divided into 17 intervals. The prediction results are obtained by the ***softmax*** function, as probabilities of belonging to a certain MIC interval. For the regression model, the objective parameter of XGboost is ***Reg: Gamma***, as MIC values can be regarded as gamma-distributed. The MICs of each sample were used as label of model training.

To prevent the XGBoost training process from generating too many trees and causing the machine learning model to be overfitted, we use fivefold cross-validation to find the most appropriate number of iterations (***num _booster_round* = *“2000”***) to the model training. In addition, using cross-validation also allows us to use as much data as possible for training, considering our small dataset. Also, the maximum depth of the tree, ***max_depth***, was set to 6, and the proportion of random sampling, ***subsample***, is 0.6.

The accuracy of the model was determined by the absolute value of the difference between the log2-transform of the predicted values and the true values.

### DNN Model Development

#### DNN

Deep learning is a concept for an approach to artificial intelligence called neural networks, and the DNN model is a basic deep learning framework. As a particular class of artificial neural networks with fully connected architecture, between the input and the output layer, there is an arbitrary number of hidden layers (Zador, 2019).

In principle, neural networks usually consist of four components: The input layer, the hidden layer(s), the output layer, and edges that connect the individual layers. More precisely, the edges connect individual nodes within the layers, whereby each transfer functions as a kind of container for a numerical value. The edges between the nodes have weights that define how the input is calculated across the edge to the next node. The arrangement of these components depends on the type and purpose of the network. Thus, the main difference between DNN and classical machine learning methods is the ability to process unstructured data through artificial neural networks (Dargan et al., 2019).

#### Model Training

**FIGURE 1:**
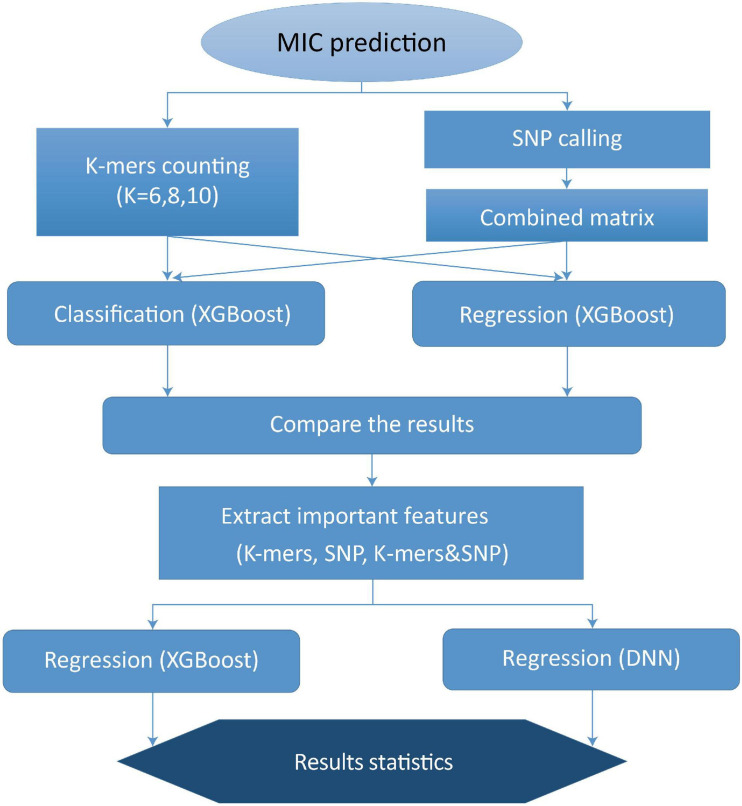
Work flow of MIC prediction modeling.

To further improve the performance of MIC prediction, we assessed the importance of *k*-mers and SNPs, respectively, based on the previous XGBoost model. We ranked all *k*-mers and SNP features using f-score as standard, and we found that the f-score values of *k*-mers and SNP features that were ranked in top 40 were greater than 1, while the others were not that significant. Thus, for the DNN method, the top 40 most important *k*-mers and SNPs were selected as features for the deep learning-based modeling. We established the following three models to predict MIC value: *k*-mers model, SNPs model, and *k*-mers & SNPs model. Our overall work flow of MIC prediction modeling is shown in Figure 1.

The DNN model with *k*-mers and SNP inputs uses a ***Dense*** neural network framework, where the top 40 most important features for predicting MIC values are fed into a 128-unit Dense layer with a ***relu*** activation function to train the DNN model. Similarly, on the test set, the absolute value of the difference between the log2 transform of the predicted value and the true value is used as the basis for assessing the accuracy of the model.

In particular, for the *k*-mers & SNPs input, we use a combined Dense + LSTM model frame. More specifically, for the top 40 characteristic *k*-mers data selected by the previous model, input the Dense layer and then input the selected top 40 feature data from the SNP site into the LSTM layer. The Dense layer and the LSTM layer are combined as the model input to train the DNN model.

## Results

**FIGURE 2:**
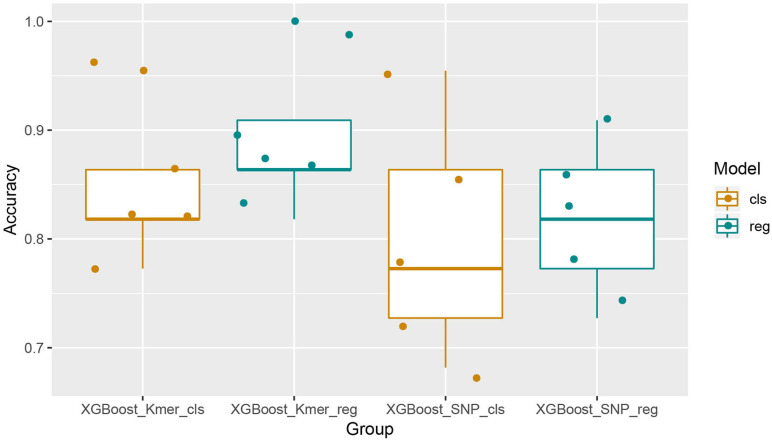
Boxplots with jittered data points of XGBoost prediction accuracies for all features. It can be seen that the results of XGBoost regression are better than the classification and that XGBoost performed better with the *k*-mers characteristics than it with SNPs.

We first used the XGBoost classification model and made five predictions using KMER (110 samples ^∗^ 559,494 *k*-mers features) and SNPs (110 samples ^∗^ 164,138 SNPs features) data. For each experiment, we set different random states from 1 to 5. Similarly, the XGBoost regression model was used to make five times predictions for both *k*-mers and SNPs data. The random states parameter was taken from 1 to 5 in order to maintain consistency in the splitting of the dataset for comparative analysis of the results. A comparison of the prediction accuracies of the models was then performed. The Boxplot grouping in Figure 2 shows the accuracy values for each of the five predictions, and Table 1 shows their mean accuracy. From these results, it is clear that the XGBoost regression model predicts better than the classification model, for both *k*-mers and SNPs data. In addition, in terms of the input feature type, XGBoost predicted *k*-mers data with better accuracy than SNPs, possibly related to the fact that SNPs is a binary input of 0 and 1. The mean predictive accuracy of the XGBoost classification model for SNPs was 0.8, while the mean accuracy of the XGBoost regression model for *k*-mers reached 0.8909091.

**TABLE 1:** Mean prediction accuracies of the XGBoost algorithm using all features of *k*-mers or SNPs (five times).

**XGBoost**	***k*-mers**	**SNPs**
Classification	0.845	0.800
Regression	0.891	0.818

**FIGURE 3:**
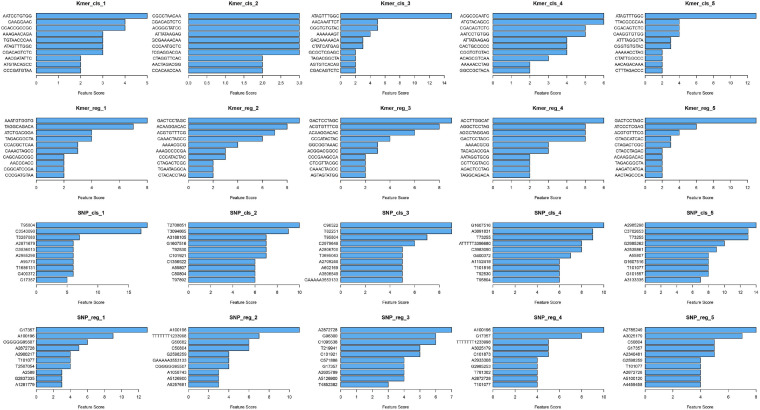
Horizontal barplots for the top 10 important features of XGBoost classification and regression models.

The top 10 important features of the classification and regression models with *k*-mers and SNPs data were statistically analyzed, respectively, and presented in the bar chart in Figure 3. As can be seen from the figure, the top 10 features of the five attempts did not completely coincide, but some common features can be found. For example, for *k*-mers’ classification model, CGACAGTCTC appears in all five runs, GACTCCTAGC appears four times in *k*-mers’ regression model, and A2872728 and G17357 also appear four times each in SNPs’ regression model.

To further optimize the model, the *k*-mers and SNPs top 40 feature datasets were taken for modeling and prediction by XGBoost regression and DNN regression, respectively. In order to enhance the reliability of the results, we used the XGBoost regression algorithm to model and predict all the features of *k*-mers and SNPs for another five times (the random_state parameter of the train_test_split function was taken from 6 to 10), and we also took their top 40 feature datasets for the XGBoost regression and DNN regression modeling. The top 40 feature datasets were also taken for the XGBoost regression and DNN regression modeling predictions.

**FIGURE 4:**
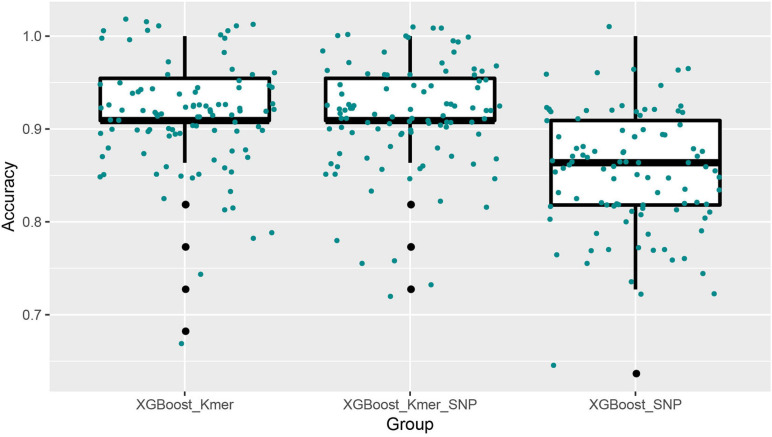
Boxplots with jittered data points of XGBoost prediction accuracies for top 40 features. Since 10 × 10 = 100 modeling predictions were made, the results of 100 predictions in each box could be seen in XGBoost’s comparison with *k*-mers, SNPs, and *k*-mers & SNPs.

**FIGURE 5:**
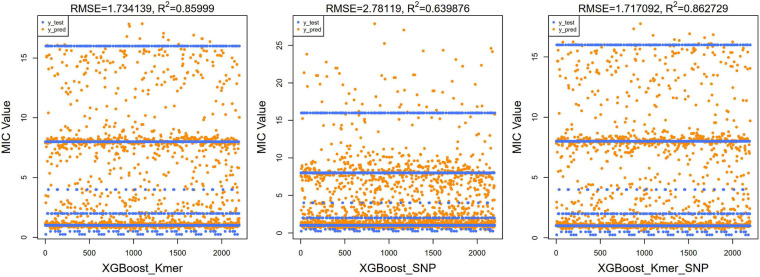
Scatter plots of true test values and predicted values of MIC using XGBoost algorithm for top 40 features (left for *k*-mers, middle for SNPs, and right for *k*-mers & SNPs). As the original *y*-value was discrete, several horizontal lines were presented in the figure. The predicted values were clustered around these lines’; RMSE and *R*^2^ values were also calculated and shown at the top of the figure.

Next, we ran the XGBoost regression model 10 times, and for the top 40 feature dataset for each experiment, we ran the XGBoost regression prediction 10 times (random states from 1 to 10). The Boxplot grouping in Figure 4 shows the accuracy values for each of the 100 predictions, and Table 2 tallies their mean values. The XGBoost regressions for *k*-mers, SNPs, and *k*-mers & SNPs data had prediction accuracies of 0.9113636, 0.8522727, and 0.9127273, with the lowest predictive accuracy for SNPs and the best for *k*-mers & SNPs. Overall, the XGBoost regression model predicted the top 40 feature dataset better than the predictions for all feature datasets, for both *k*-mers and SNPs (Tables 1, 2). We show the *y*-test and *y* predicted values for all 100 predictions and see that the predicted values largely fluctuate around the true values (Figure 5).

**TABLE 2:** Mean prediction accuracies of the XGBoost algorithm using top 40 features of *k*-mers or/and SNPs (10 × 10 times).

**XGBoost (Top 40)**	***k*-mers**	**SNPs**	***k*-mers & SNPs**
Regression	0.911	0.852	0.913

**FIGURE 6:**
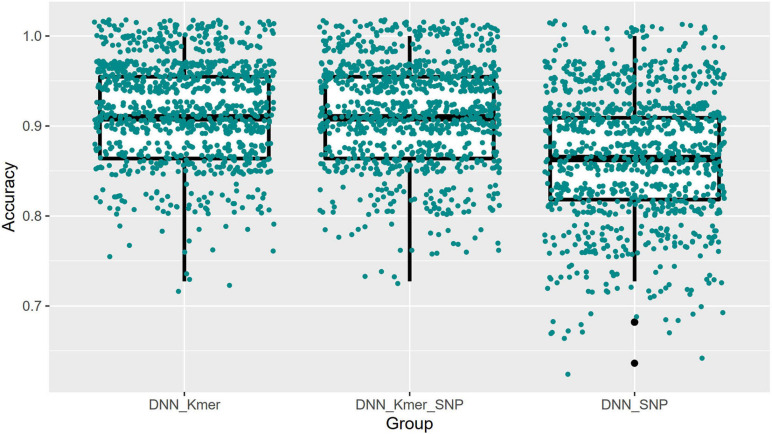
Boxplots with jittered data points of DNN prediction accuracies for top 40 features (100 × 10 times training).

**FIGURE 7:**
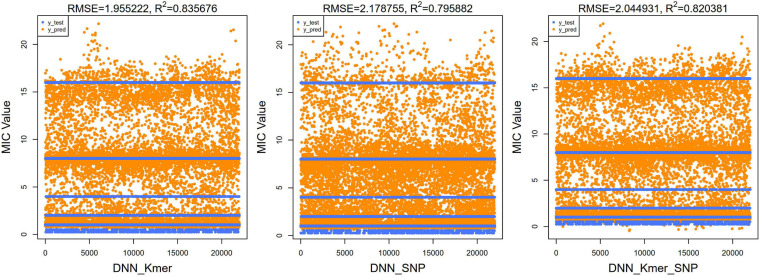
Scatter plots of true test *y*-values and predicted *y*-values using DNN algorithm for top 40 features (100 × 10 times training). The predicted values were clustered around these lines; RMSE and *R*^2^ values were also calculated and shown at the top of the figure.

Similarly, for the DNN model, the top 10 important features selected by XGBoost were trained for a total of 100 times of random resolution, respectively. The Boxplot grouping in Figure 6 shows the accuracy values of 1,000 times of prediction, and their average values are calculated in Table 3, and the test and predicted values for all 1,000 predictions are shown in Figure 7. Regressions for *k*-mers, SNPs, and *k*-mers & SNPs had prediction accuracies of 0.9189091, 0.8705455, and 0.9177273, respectively, with the lowest prediction accuracy for SNPs and very similar prediction accuracies for *k*-mers and *k*-mers & SNPs, all of which were relatively high.

**TABLE 3:** Mean prediction accuracies of the DNN algorithm using top 40 features of *k*-mers or/and SNPs (100 × 10 times training).

**DNN (Top 40)**	***k*-mers**	**SNPs**	***k*-mers & SNPs**
Regression	0.919	0.871	0.918

For regression models, the mean square root of the error between the predicted and true values (RMSE) is usually used as a model evaluation metric, and the coefficient of determination (*R*^2^) is used to indicate how well the model predicts the true value compared to the mean value model. We calculated the RMSE and *R*^2^ values of our XGBoost and DNN models. For our XGBoost models, the RMSE values were 1.734, 2.781, and 1.717, and *R*^2^ values were 0.860, 0.640, and 0.863, respectively (Figure 5). The RMSEs of the DNN models were 1.955, 2.179, and 2.045 and the *R*^2^ values of the DNN model were 0.836, 0.796, and 0.820 (Figure 7). *R*^2^ is an indicator used in regression models to evaluate the degree of agreement between the predicted value and the actual value, with a maximum value of 1. It can be seen that, overall, our models fit well.

In summary, our analysis showed that the XGBoost classification model reached over 80% prediction accuracy, and the model with *k*-mers data gave better results than SNPs inputs. Compared with the XGBoost classification model, the overall performance of the XGBoost regression model is improved (89.1 and 81.8% for *k*-mers and SNPs data, respectively). The MIC value is continuously distributed, and the effect of the regression model may be more realistic. DNN neural network models perform better in predicting MIC values with improved overall accuracy compared to XGBoost models. On the other hand, the *k*-mers and SNPs top 40 feature dataset was sufficient to obtain good prediction results (above 85% accuracy), with *k*-mers and mixed *k*-mers & SNPs features performing well and the DNN regression model performing better than the XGBoost regression approach.

## Discussion

Based on metagenomic data, in this study, sequence analysis was used to obtain SNPs information and nucleotide *k*-mers count information queue data; machine learning and deep learning methods were then applied to establish a prediction model for the MIC value of *K. pneumoniae*. By feature selection, we proposed a top 40 feature-based regression model, which had the best predictive performance of 91%.

First, according to Naha et al. (2021) and Okanda et al. (2020), we found that gene mutations may affect drug resistance of *Klebsiella*; thus, we tried to find the relevant sites affecting resistance by calling SNPs. After pre-processing the raw data by using biogenetics tools BWA, BCFTools, and SamTools, we obtained a matrix of mutation site and sample list. We took the mutated gene site as the features and built the machine learning model of classification and regression, respectively. We used 110 samples for prediction, and the prediction results above show that the mean accuracy of the SNPs classification model was 80% and the mean accuracy of the SNPs regression model was 81.81%, which shows that the performance of the regression model is better than the multi-classification model. Then, based on the method previously described by Nguyen et al. (2019), we created both XGBoost classification and regression models using *k*-mers counts as input features, respectively, and made MIC predictions for 110 samples. As described above, after five runs, we obtained a mean accuracy of 84.54% for the *k*-mers classification model and 89.09% accuracy for the *k*-mers regression model. This result again shows that the multi-classification model does not perform as well as the regression model. In addition, the prediction of MIC values using SNPs loci was less effective than that of *k*-mers prediction, which may be due to the fact that the input to the SNPs is binary data with only mutated (labeled as 1) and unmutated (labeled as 0) features, while the input to the *k*-mers counting model are continuous variables, making it more effective for regression model training.

To evaluate our model, we compared MIC prediction models built by related studies. In the study by ValizadehAslani et al. (2020), the authors used the XGBoost model with *k*-mers features, and the result shows an accuracy of around 91% in predicting the MIC value of meropenem against *K. pneumoniae*, which was close to our results. Another study by Nguyen et al. (2019) also used the XGBoost model to predict MICs for non-typhoidal *Salmonella*, resulting in an average accuracy of 90% without a large number of samples. We decided to try more advanced deep learning approach for prediction. As the K-mers and SNPs had too many feature values, and the neural network could not accept features with too high dimensions, we selected some of important features as the training data to avoid overfitting.

The XGBoost regression model gives a score of importance for each feature during the training process. We selected the top 40 highest scores from the *k*-mers and the SNPs regression model, respectively, and then we used these total 80 important features as a new dataset, to predict MIC values using both XGBoost and DNN algorithms. In consideration of training time and server capacity, we only use regression models for prediction.

Comparing the results in Tables 2, 3, the DNN model performs better than the classical XGboost machine learning approach in predicting MIC values, with a slight improvement in both accuracy rates. However, the reason for the small improvement may be due to the fact that only important features were selected for training and the overall amount of sample size was relatively small. In addition, the prediction accuracy of the model improved by combining the significant features of *k*-mers and SNPs to produce a new dataset than training with a single type of feature.

We found the annotated.gff file of the reference genome from NCBI and the paper on the whole gene analysis of the reference genome HS11286 by the team of Liu (Liu et al., 2012); the *K. pneumoniae* resistance genes were found from this paper and we identified loci belonging to these gene fragments from important features in the SNPs model. The pKPHS3 was mentioned in the study (Liu et al., 2012) as possessing 13 important resistance determinants, such as tetG, cat, sul1, dfra12, aac(3)-Ia, and aph. Genes were found among the important features of our SNPs, such as site T37808, which belongs to the tetG gene family, an important gene family that influences tetracycline resistance. This demonstrates that the important feature values obtained from our model training may help us to understand the reasons for the development of resistance, and why there are anti-tetracycline resistance genes present due to the presence of tra isoconjugate transfer genes in pKPHS2 and pKPHS3, which is the type of gene that causes resistance to spread between genera (Liu et al., 2012). Moreover, meropenem belongs to the class of beta-lactam antibiotics, which are classified as carbapenems. According to Reyes et al. (2019), the most common resistance mechanism of *K. pneumoniae* to carbapenem antibiotics is the production of enzymes with carbapenemase activity, which hydrolyze beta-lactam antibiotics, while we also identified mutations in the beta-lactamase gene from important features in SNPs models, such as C1114518 and G1114674; i.e., mutations in the beta-lactamase gene may be responsible for the high MIC values.

In summary, we found that there are still a lot of genes in *Klebsiella* that belong to hypothetical proteins, and the loci we derived from this study can help to annotate and study these hypothetical proteins. Furthermore, in clinical practice, deep learning-based modeling and prediction by selecting important feature values can significantly improve detection efficiency compared to experimental methods of measuring MIC values, providing doctors with a faster access to information on patient resistance for drug administration and improving the effectiveness of antibiotic use, enabling patients to receive medication promptly. It also reduces the cost of the experiment.

## Additional Information

CentOS Linux release 7.2.1511 (Core)

Linux version 3.10.0-327.el7.x86_64 (builder@kbuilder.dev.centos.org) (gcc version 4.8.3 20140911 (Red Hat 4.8.3-9) (GCC)

jupyter lab version 0.34.9

Python 3.7.2

## Data Availability Statement

The metagenomic sequence data included in this study can be found in the NCBI SRA (BioProject accession numbers PRJNA376414, PRJNA386693, and PRJNA396774).

## Author Contributions

JG and JL conceived ideas and designed the study. AY, JG, XJW, and XLW wrote the manuscript. ZML and RJ performed the bioinformatics analysis. RT and ZQL constructed the machine learning models. All authors read or revised the manuscript and approved the final version.

## Conflict of Interest

JG, AY, RT, XJW, and RJ are employed by Shanghai Biotecan Pharmaceuticals Co., Ltd. and Shanghai Zhangjiang Institute of Medical Innovation. The remaining authors declare that the research was conducted in the absence of any commercial or financial relationships that could be construed as a potential conflict of interest.

## Publisher’s Note

All claims expressed in this article are solely those of the authors and do not necessarily represent those of their affiliated organizations, or those of the publisher, the editors and the reviewers. Any product that may be evaluated in this article, or claim that may be made by its manufacturer, is not guaranteed or endorsed by the publisher.

## Footnotes

1
https://www.ncbi.nlm.nih.gov/assembly/GCF_000240185.1
